# Dental high-speed handpiece and ultrasonic scaler aerosol generation levels and the effect of suction and air supply

**DOI:** 10.1017/ice.2022.196

**Published:** 2023-06

**Authors:** Joanne Jung Eun Choi, Jason Chen, Yunsun Jane Choi, Susan M. Moffat, Warwick J. Duncan, J. Neil Waddell, Mark Jermy

**Affiliations:** 1 Sir John Walsh Research Institute, Faculty of Dentistry, University of Otago, Dunedin, New Zealand; 2 Department of Mechanical Engineering, University of Canterbury, Christchurch, New Zealand

## Abstract

**Objective:**

Exposure to aerosol spray generated by high-speed handpieces (HSHs) and ultrasonic scalers poses a significant health risk to oral health practitioners from airborne pathogens. Aerosol generation varies with different HSH designs, but to date, no study has measured this.

**Materials and methods:**

We measured and compared aerosol generation by (1) dental HSHs with 3 different coolant port designs and (2) ultrasonic scalers with no suction, low-volume evacuation (LVE) or high-volume evacuation (HVE). Measurements used a particle counter placed near the operator’s face in a single-chair, mechanically ventilated dental surgery. Volume concentrations of aerosol, totaled across a 0.3–25-µm size range, were compared for each test condition.

**Results:**

HSH drilling and scaling produced significantly high aerosol levels (*P* < .001) with total volume concentrations 4.73×10^8^µm^3^/m^3^ and 4.18×10^7^µm^3^/m^3^, respectively. For scaling, mean volume of aerosol was highest with no suction followed by LVE and HVE (*P* < .001). We detected a negative correlation with both LVE and HVE, indicating that scaling with suction improved operator safety. For drilling, simulated cavity preparation with a 1-port HSH generated the most aerosol (*P* < .01), followed by a 4-port HSH. Independent of the number of cooling ports, lack of suction caused higher aerosol volume (1.98×10^7^ µm^3^/m^3^) whereas HVE significantly reduced volume to −4.47×10^5^ µm^3^/m^3^.

**Conclusions:**

High concentrations of dental aerosol found during HSH cavity preparation or ultrasonic scaling present a risk of infection, confirming the advice to use respiratory PPE. HVE and LVE both effectively reduced aerosol generation during scaling, whereas the new aerosol-reducing ‘no air’ function was highly effective and can be recommended for HSH drilling.

In dentistry, the high-speed handpiece (HSH) is used for removing tooth structure to prepare teeth for restoration.^1^ Most modern HSHs incorporate air or air–water coolant ports, designed to spray water to improve cutting and polishing efficiency while minimizing pulp injury.^2^ The water coolant and the rotary cutting bur generate aerosol which, when combined with oral fluids creates bioaerosol.^2^ (Bio)aerosols may remain in the air for protracted periods, and they have the potential to transmit respiratory infections to oral health practitioners.^3,4^

The ongoing coronavirus disease 2019 (COVID-19) pandemic has recorded >476 million cases and >6.1 million deaths so far.^5^ Oral health practitioners face the greatest risk of contracting COVID-19 from aerosol exposure, more so than nurses and general physicians,^6–8^ and the WHO recommends that oral health clinicians employ strict personal protection measures to avoid or minimize aerosol-producing procedures.^9^ Guidelines on the provision of dental services during the COVID-19 pandemic have been frequently updated.^8,9^ Policy documents focus on dental instruments as aerosol sources and recommend rubber-dam and high-volume evacuation (HVE) suction as mitigating measures.^2,4,9,11,12^ Severe acute respiratory coronavirus virus 2 (SARS-CoV-2) is the primary current concern, but our findings apply to many airborne pathogens.

Depending on the design, HSHs generate different levels of aerosol.^1,13^ For example, air-turbine HSHs allow rapid preparation of dental hard tissues with minimal pulpal damage, but they create considerable aerosol.^14,15^ Conversely, electric-motor HSHs offer constant power and rarely stall, potentially creating less bioaerosol. Some dental HSHs include a function that directs air onto the cutting surface to help cool the tooth, disperse water spray, and clear debris.^15,16^ This ‘chip air’ function produces higher aerosol levels but can be deactivated in newer handpieces. Currently, few reports have quantified how much aerosol is produced by different types of HSHs, including those with or without the ‘chip air’ function.^15^ Furthermore, the literature shows that ultrasonic scalers produce ∼3 times the bioaerosol compared to hand instruments.^12,17^

To reduce aerosol production and to provide a drier operation field during dental procedures, low-volume evacuation (LVE) or high-volume evacuation (HVE) suction systems may be employed.^17,18^ Evaluating the effectiveness of different types of suction systems for drilling and scaling is important in developing standard guidelines for oral health practitioners to minimize cross infection by airborne pathogens.

In this study, we compared aerosol generation (1) in dental HSHs with 3 different coolant port designs (1, 3, or 4 ports), with and without the new aerosol-reducing function (ie, water jet only, no ‘chip air’) and (2) in ultrasonic scalers operated under 3 different suction conditions (ie, no suction, low-volume evacuation [LVE], or HVE). We formulated 2 null hypotheses: (1) There is no significant difference in aerosol levels generated between dental HSH with the 3 different coolant-port designs and (2) there is no significant differences in aerosol levels generated during ultrasonic scaling with the 3 different suction conditions.

## Materials and methods

### Particle concentration measurements

Tests were conducted using a dental mannequin in an enclosed, windowless dental surgery. The floor space measured ∼3.9 m × 3.5 m × 2.7 m. The room was mechanically ventilated (supply at 0.042 m^3^/s or 4.1 air changes per hour [ACH]). Air entered the room through a ceiling grille above the foot of the dental chair and passively exhausted through a ceiling grille above the head of the dental chair. To reduce background particle concentration, all surfaces (eg, floor) were cleaned, and operators wore clean personal protective equipment (PPE). A Sheffield HEPA-13 air purifier (Prolink, Auckland, NZ) was run at maximum fan speed, with a clean air delivery rate of 320 m^3^ per hour (the equivalent of an additional 8.6 ACH). The purifier was run for 40 minutes before the sequence of measurements commenced, and it ran continuously throughout the measurements at maximum fan speed. The air filtration rate, being twice the ventilation rate, had a significant effect on the background particle concentrations but was not expected to significantly affect the concentration at the operators’ location. The concentration of particles here is dominated by the production of aerosol at the patient’s mouth, and the particles are measured within seconds of their generation.

Particle concentrations were measured with 2 AeroTrak particle counters (TSI, Shoreview, MN, USA): (1) an AeroTrak 9306 (with isokinetic inlet, TSI part no. 700003) mounted on a tripod at roughly the position of the dental practitioner’s face and (2) an AeroTrak 9310 (with isokinetic inlet 700068) placed on a bench located along the wall opposite the dental chair. Both counters independently measured particle concentrations in 6 size ranges: 0.3–0.5 µm, 0.5–1.0 µm, 1.0–3.0 µm, 3.0–5.0 µm, 5.0–10.0 µm, and 10.0–25.0 µm. A Kestrel 5000 Environmental Meter (Nielsen-Kellerman, Boothwyn, PA, USA) logged temperature and humidity at 1-minute intervals. The Dental Council of New Zealand does not normally specify ventilation rates for dental surgeries, but it did release some guidance during COVID-19 alert level 2 in 2021. (This was the second of 4 alert levels, and level 2 applied during periods when there was a low rate of community transmission.) This guidance stated that rooms with 1–2 ACH were to be considered poorly ventilated and that high-volume suction was to be considered essential in such rooms. It specified stand-down periods after aerosol-generating procedures of 10–30 minutes depending on what combinations of evacuation and dental dams were used. The net ventilation rate of 8.6 ACH in our test room is high for most mechanically ventilated buildings, but it falls within ASHRAE recommendations of ≥6 ACH for most treatment rooms other than operating theatres. We considered that, given that the building ventilation supply is filtered, 8.6 ACH provided an acceptably low level of background particle concentration for these tests.

### Data processing and classification of activities

Various oral health activities simulated by an operator and assistant were classified into 17 types: ‘consultation: talking, no purifier’; ‘consultation: silence, no purifier’; ‘triplex, no purifier’; ‘movement of people (pax) (simulating a patient/person), no purifier’; ‘lunch, no purifier’; ‘preparation, no purifier’; ‘consultation: talking’; ‘consultation: silence’; ‘triplex’; ‘movement of pax’; ‘purifier ON’; ‘scaling’; ‘rest (persons present with minimal movement)’; ‘preparation’; ‘drilling’; ‘test of handpiece setting’; and ‘room empty.’ Suction was classified into HVE suction, LVE suction, or no suction. For drilling, 4 location classifications were used: upper incisor, upper left molar, lower front incisor, patient’s right, or lower right molar. Also, 3 drilling directions were used: rear, front, or occlusal. Finally, 3 handpiece configurations were used: ‘1 port, water and spray, NSK Z85 at maximum air pressure,’ ‘4 ports, water and spray, NSK Z95L at maximum air pressure,’ or ‘4 ports, water, NSK Z95L at maximum air pressure and no spray.’

### Calculation of excess total particle volume concentration

The AeroTrak instruments accumulated particle counts in 6 size ranges (channels 1–6): 0.3–0.5 µm, 0.5–1.0 µm, 1.0–3.0 µm, 3.0–5.0 µm, 5.0–10 µm, and 10–25 µm. Assuming that all measured particles were spherical with a uniform size distribution, the volume mean diameters for each channel were 0.42 µm, 0.83 µm, 2.4 µm, 4.2 µm, 8.3 µm, and 20 µm, respectively. The particle concentration (number per unit volume) in each channel was multiplied by the volume mean diameter for that channel to calculate the particle volume per unit volume of air sampled. These data were summed over all 6 channels to calculate total particle volume per unit volume of air sampled.

In our experiment, a persistent background level of particles was not generated by dental treatment activity (ie, mainly shed skin cells, clothing fibers, etc). During the ‘rest’ activities, the concentrations measured by the Aerotrak 9310 instrument (at a distance from the practitioner) corresponded well with the Aerotrak 9306 instrument (near the practitioner). Therefore, the Aerotrak 9310 measurements were assumed to be equal to the background aerosol concentration. Minute differences between the 2 instruments during these rest periods were attributed to the noise inherent in particle counting and the spatial nonhomogeneity of the air currents. The AeroTrak 9310 data were subtracted from the AeroTrak 9306 data to obtain the excess aerosol concentrations released in the vicinity of the dental practitioner by the activities of interest. The mean over each consecutive 30-second counting interval during each activity of interest was calculated. The mean over the repeated measurements of the same type of activity was then calculated.

### Dental operative procedures

Two operators (1 clinician and 1 assistant) were present, dressed in minimum PPE requirements (surgical mask to ASTM F2100 standard, eye protection, gloves, and outer protective clothing or gown) according to the DCNZ COVID-19 level 1 guidelines.^11^

**Fig. 1.**
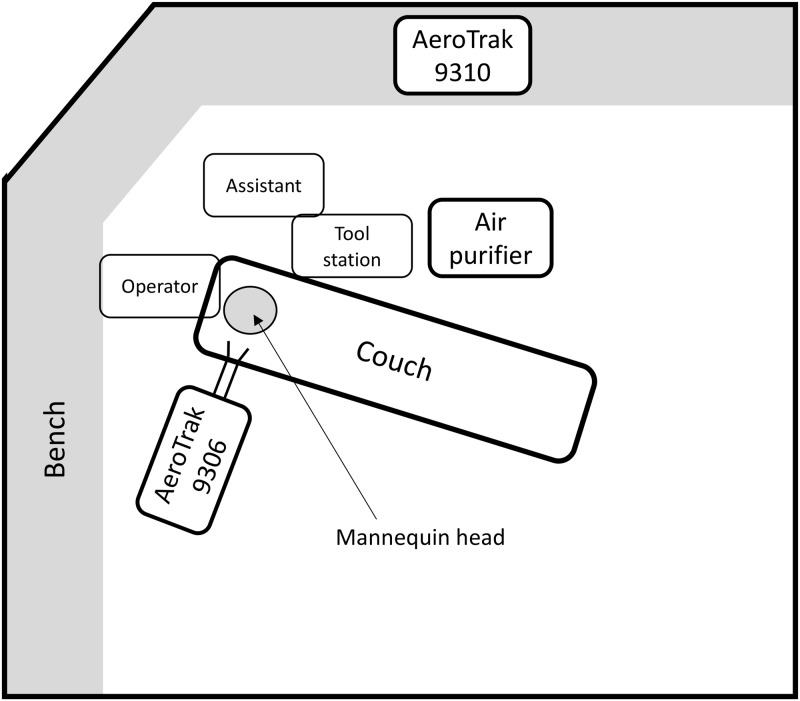
Dental test room layout.

The clinician performed the ultrasonic scaling and drilling operative procedures following the same sequence for each test (Fig. 1). All simulated dental operative procedures were conducted using a Viva Ace portable dental unit (NSK, Japan) and simulation teeth (PRO2001-UL-SC-FEM-32, Nissin, Japan). Teeth sets were attached to a Nissin type 1 dental simulator head set (including head, type 1 articulator, and small mask) mounted on the dental chair. The ultrasonic scaler and HSHs used are listed in Table 1.

**Table 1.** List of Dental Equipment Used in the Current Study

Equipment	Brand Name (NSK, Japan)	No. of Cooling Ports	Chip-Air Function
High-speed handpiece (electrical)	Ti-Max Z45L	1	Yes
	Ti-Max Z95L	4	Yes
Ultrasonic scaler	Varios2 VA2-LUX	…	…

**Fig. 2.**
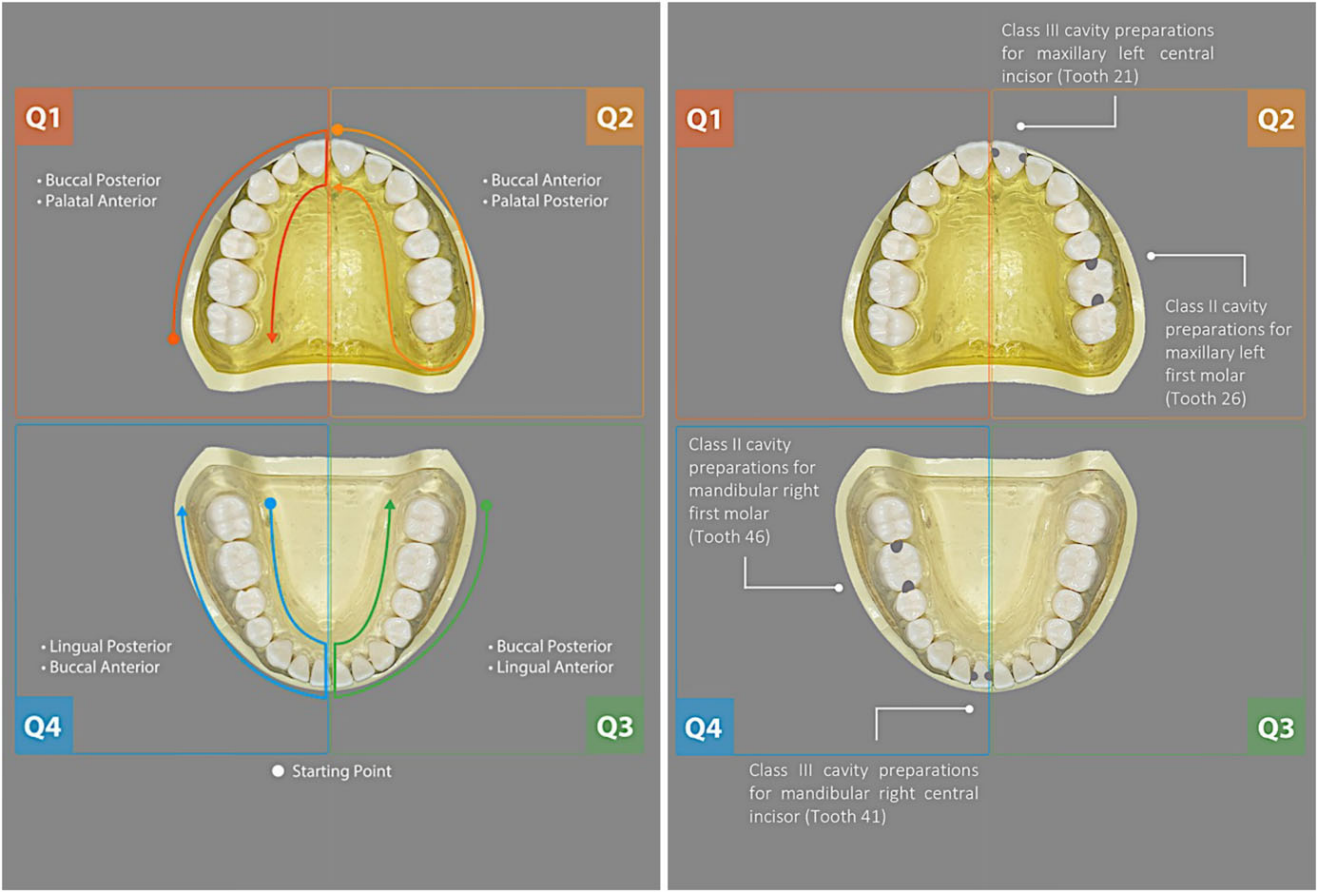
Diagrams showing the sequence of ultrasonic scaling for each quadrant (left) and drilling for Class II and III preparations (right). Q1: quadrant 1, patient’s upper right; Q2: quadrant 2, patient’s upper left; Q3: quadrant 3, patient’s lower left; Q4: quadrant 4, patient’s lower right. Dots in the first figure (left) indicate the starting point of ultrasonic scaler and the arrow indicates the direction and the finishing point. Gray areas highlighted on the teeth in the second figure (right) indicate the location of cavity preparations.

For ultrasonic scaling, each test was carried out with a Varios2-LUX scaler (NSK) with a type G6 tip and the recommended G8 (80%) power setting. Scaling started from the distal side of the last molar in quadrant 1 (Q1, patient’s right), continuously moving to Q2, Q3, and Q4 for 2 minutes per quadrant (Fig. 2). Each test was repeated using no suction and 2 different types of suction tip: LVE suction (saliva ejector clear blue tip, Henry Schein, Melville, NY, USA) and HVE suction (206P HVE tubes, Premium Plus, Bournemouth, UK). Following standard clinical protocols, the LVE suction tip was placed at the posterior region of the last molar and remained there throughout the procedure. The saliva ejector is designed to be used by a single operator, which was simulated in the current study.

During scaling with HVE suction, the assistant positioned the suction tip to continuously follow ∼1 cm behind the scaler as indicated in Figure 2 and Table 2. For drilling, a class III cavity was prepared for a maxillary left central incisor (FDI notation tooth 21) and a mandibular central incisor (tooth 41). A class II cavity was prepared for the maxillary left first molar (tooth 26) and mandibular right first molar (tooth 46). A Diamond Cylinder bur (841F 314 012 Fine/5, Ivoclar Vivadent, Schaan, Liechtenstein) was used with each HSH, with each set repeated 5 times (Table 1). Also, 10 surfaces in total were drilled per set per test including the mesial or distal part of the tooth (Table 2).

**Table 2.** Summary of Dental Drilling Protocols and Sequence

Class III cavity	Tooth 21 (upper anterior)		
Set 1 #1 (2 min)	MP	No suction	4 ports water + spray
Set 1 #2 (2 min)	MI	No suction	4 ports water + spray
Set 1 #3 (2 min)	DP	HVE	4 ports water + spray
Set 1 #4 (2 min)	DI	HVE	4 ports water + spray
Rest (∼2 min that include replacing tooth, refilling water, and changing handpiece)			
#1–4 repeated ×5 (5 sets completed)	Same as above	Same as above	1 port 4 ports with water + spray 4 ports with water only
**Class III cavity**	**Tooth 41 (lower anterior)**		
Set 1 #1 (2 min)	ML	No suction	4 ports water + spray
Set 1 #2 (2 min)	MI	No suction	4 ports water + spray
Set 1 #3 (2 min)	DL	HVE	4 ports water + spray
Set 1 #4 (2 min)	DI	HVE	4 ports water + spray
Rest (∼2 min that include replacing tooth, refilling water, and changing handpiece)			
#1–4 repeated ×5 (5 sets completed)	Same as above	Same as above	1 port 4 ports with water + spray 4 ports with water only
**Class II cavity**	**Tooth 26 (upper posterior)**		
Set 1 #1 (2 min)	MO	No suction	4 ports water + spray
Set 1 #2 (2 min)	DO	No suction	4 ports water + spray
Set 1 #3 (2 min)	MO	HVE	4 ports water + spray
Set 1 #4 (2 min)	DO	HVE	4 ports water + spray
Rest (∼2 min that include replacing tooth, refilling water, and changing handpiece)			
#1–4 repeated ×5 (5 sets completed)	Same as above	Same as above	1 port 4 ports with water + spray 4 ports with water only
**Class II**	**Tooth 46 (lower posterior)**		
Set 1 #1 (2 min)	MO	No suction	4 ports water + spray
Set 1 #2 (2 min)	DO	No suction	4 ports water + spray
Set 1 #3 (2 min)	MO	HVE	4 ports water + spray
Set 1 #4 (2 min)	DO	HVE	4 ports water + spray
Rest (∼2 min that include replacing tooth, refilling water, and changing handpiece)			
#1–4 repeated ×5 (5 sets completed)	Same as above	Same as above	1 port 4 ports with water + spray 4 ports with water only

Note. MP, Mesial Proximal; MI, Mesial Incisal; DP, Distal Proximal; DI, Distal Incisal; ML, Mesial Lingual; DL, Distal Lingual; MO, Mesial Occlusal; DO, Distal Occlusal; HVE, High-volume evacuation.

**Table 3.** The Mean Level of Aerosol (Volume of Particles) Measured in Different Settings and Variables

Experimental Specification	Variable	Mean Total Volume of Particles (*µ*m^3^/m^3^ ± SD)
Mean level of aerosol (volume of particles) measured during 8 activities recorded	Activities	
	Drilling	4.73 × 10^8^ ± 7.74 × 10^7^
	Scaling	4.18 × 10^7^ ± 1.22 × 10^7^
	Consultation, talking	−8.59 × 10^6^ ± 2.39 × 10^6^
	Consultation, silence	−6.93 × 10^5^ ± 2.32 × 10^6^
	Triplex	−3.39 × 10^6^ ± 1.48 × 10^6^
	Rest	1.98 × 10^7^ ± 5.00 × 10^6^
	Preparation	1.75 × 10^6^ ± 2.53 × 10^6^
	Movement of Pax	−4.28 × 10^6^ ± 8.32 × 10^5^
Mean amount of aerosol (volume of particles) measured during scaling and drilling with different suction types and different types of high-speed handpieces with different number of cooling ports and with and without suction	Activities	
	Scaling, no suction	8.06 × 10^4^ ± 1.21 × 10^4^
	Scaling, LVE suction	1.73 × 10^4^ ± 3.96 × 10^3^
	Scaling, HVE suction	−2.04 × 10^2^ ± 4.06 × 10^2^
	Drilling, 1 port	1.19 × 10^6^ ± 1.08 × 10^5^
	Drilling, 4 ports	2.34 × 10^6^ ± 1.63 × 10^5^
	Drilling, 4 ports no air	1.58 × 10^5^ ± 2.35 × 10^4^
	Drilling, no suction	1.98 × 10^7^ ± 3.82 × 10^7^
	Drilling, HVE suction	−4.47 × 10^5^ ± 1.43 × 10^5^
Mean amount of aerosol (volume of particles) measured at 4 different sites during drilling procedure	By location	
	Upper anterior	1.64 × 10^6^ ± 1.48 × 10^5^
	Upper posterior	7.27 × 10^5^ ± 8.89 × 10^4^
	Lower anterior	1.68 × 10^6^ ± 1.60 × 10^5^
	Lower posterior	6.28 × 10^5^ ± 7.47 × 10^4^

Proximal and/or occlusal teeth surfaces are more susceptible to dental caries than smooth (eg, buccal, labial, lingual, or palatal) surfaces. The central incisor was chosen to demonstrate maximum aerosol generation because this tooth has the least soft-tissue barrier. A class II cavity preparation (proximal and occlusal surfaces) on the first molar was chosen because this tooth has a higher caries incidence among adults.^19,20^ The HSH speed was set at 40,000 revolutions per minute. The 4-port HSH had a minimum water consumption rate of 37 mL per minute and minimum ‘chip air’ rate of 2 mL per minute. The whole test was performed first with HVE and then without suction. Maximum suction was used with the tip kept close to the cavity being prepared.

### Statistical analyses

Dental aerosol levels generated during different operative procedures (section 2.4) were compared using the Kruskal-Wallis and post hoc tests. Dental aerosol particle volume levels were compared using the Mann-Whitney U test. Statistical analyses were conducted using SPSS version 27 software (IBM, Armonk, NY, USA) with statistical significance set at *P* < .05. The effects of the dental operative procedures and the corresponding variables were also investigated using the Pearson correlation coefficient (R value). Because the signal noise in the measurements is considerable, R values of >0.1 and <−0.1 were interpreted as evidence of correlation.

## Results

**Fig. 3.**
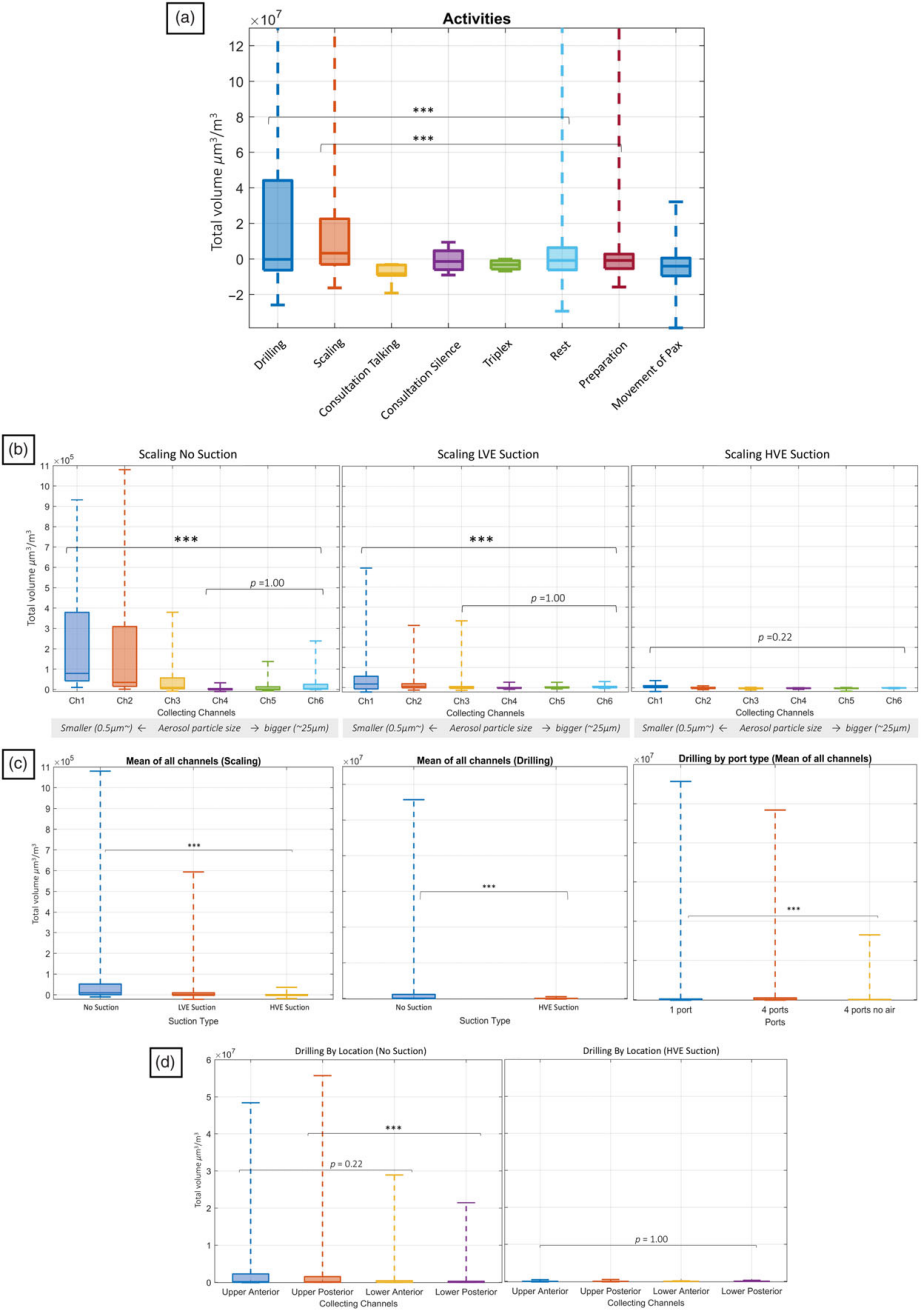
The total volume of aerosol (µm^3^/m^3^) created by (a) different activities; (b) during scaling and drilling with different types of suction; and (c) during scaling and drilling with different types of suction [the volume mean diameters for each size range are 0.42 µm (channel 1), 0.83 µm (channel 2), 2.4 µm (channel 3), 4.2 µm (channel 4), 8.3 µm (channel 5), and 20 µm (channel 6)]; and (d) during drilling in different locations; 1 incisor and 1 posterior tooth per maxillary and mandibular arch, with and without HVE.

Drilling and scaling generated a total aerosol volume of 4.73(±0.774)×10^8^ µm^3^/m^3^ and 4.18(±1.22)×10^8^ µm^3^/m^3^, respectively, which were significantly higher (*P* < .001) than those generated with other activities, for example, by talking during the patient–clinician consultation, using the triplex air-water syringe, or during rest periods (Table 3 and Fig. 3A).

For scaling, the mean aerosol particle volume recorded was significantly higher, with no suction (8.06±1.21×10^4^ *µ*m^3^/m^3^), with the least aerosol generated when HVE was used (*P* < 0.001) (Table 3 and Fig. 3C). For the scaling activities, a negative correlation (reduced risk-associated variables) was found with both low- and high-volume suction (Fig. 3C). When each particle size range was compared separately for scaling with the different suction systems, HVE reduced the aerosol level significantly compared to LVE or no suction, and there was no significant difference in the total volume of smaller or bigger particles detected (*P* = .01) (Fig. 3B).

Simulated cavity preparation (drilling) with the 1-port HSH showed the highest aerosol level (*P* < 0.01), followed by drilling with the 4-port handpiece and 4-port handpiece with no air function (Table 3 and Fig. 3C). Regardless of the number of cooling ports, suctionless drilling resulted in a higher aerosol volume, 1.98(±3.82)×10^7^ *µ*m^3^/m^3^, whereas the particle volume was reduced significantly with HVE (−4.47(±1.43)×10^5^ *µ*m^3^/m^3^). The mean level of aerosol produced by drilling (with no suction) was also influenced by the location of cavity preparation (Fig. 3D), but we did not detect significant differences either between maxillary and mandibular teeth preparations or between anterior and posterior teeth (*P* = 1.00) (Fig. 3D). Correlation analysis, however, revealed a negative correlation for posterior teeth, drilling with a 4-port handpiece with ‘chip air’ deactivated or drilling with any number of ports with HVE. A positive correlation (riskier variables) was reported with drilling maxillary incisors, using a 1- or 4-port HSH with normal spray function and with drilling with any type of high-speed handpiece without suction.

## Discussion

We investigated the effect of suction and air supply on aerosol generation during dental drilling and scaling to stratify risk from different combinations of variables for each procedure and enable guidance for oral health practitioners treating patients during the COVID-19 pandemic or considering other airborne pathogens. The null hypotheses were rejected because drilling and scaling with LVE or HVE suction reduced aerosol generation significantly. We detected significant reductions in aerosol production when drilling was done with a HSH with more coolant ports, and particularly those handpieces with the new aerosol-reducing ‘no chip air’ function.

To investigate the different combination of variables for generating aerosol in dental operative procedures, we preferred the air sampling method over the ‘settle plate’ methodology. Although most dental aerosol studies have utilized the ‘settle plate’ method,^12^ it only detects what has fallen onto a surface and is thus limited to detecting larger droplets. Air sampling provides more clinically relevant data, enabling investigators to detect both aerosol and airborne droplets before they have fallen to the ground.^12,21–24^ The difficulties of comparing results of previous dental aerosol studies included a lack of consistent methodology and inadequate sampling details (eg, time, frequency of air sampling, distance from sources, etc). In the current study, we have provided a standardized protocol, and our findings can act as a baseline reference for other variables of interest. Because the focus of the current study was to measure and compare the effect of different suctions and air supplies for dental high-speed handpieces and ultrasonic scaling, only 1 brand or type per variable was investigated, which could be a limitation of the study. Further work should include a variety of types (and brands) of handpieces, scalers, and suction systems to investigate their relative ability to reduce aerosol level under identical in vivo clinical conditions. A disadvantage of or approach is that it does not test the effect of other precautions (eg, screening patients for infectious diseases, PPE, mouthwash).

Our finding that high-speed handpieces and ultrasonic scaling produce the most concentrated dental aerosol aligns well with the current literature.^12,25^ Ultrasonic scalers and the efficiencies of different suction types, in particular, have been topical in the current pandemic. Dental hygienists traditionally provide care with the aid of a dental assistant, due to the bulkiness of the high-volume suction (HVE) and difficulties in maneuvering it. Dental hygienists working solo prefer using the LVE (saliva ejector) suction system, which is less bulky and easier to use than HVE suction.^17,26^ Moreover, LVE saliva ejectors provide an easy means of clearing the operating field and better patient comfort than HVE suction.^18^

Many clinical guidelines recommend 4-handed dentistry during the COVID-19 pandemic and that HVE be used during all dental treatment to minimize aerosol production.^11,27–29^ Other guidelines state that aerosol production by dental handpieces is complex and dependent on multiple factors such as handpiece speed, mix of air and water, coolant ports and types of bur used,^27,28^ which have been investigated in the current study.

Our results show that both HVE and LVE significantly reduced the aerosol level for scaling. Whereas HVE was very efficient in reducing aerosol particles all sizes to a minimum level, LVE was more effective in the larger particle range. This finding is consistent with previous studies^30–32^; however, it disagrees with the findings from Matys and Grzech-Lesinka.^14^ Interestingly, there was a statistically significant difference in aerosol particle levels between scaling with HVE and LVE. This contradicts the findings of Holloman et al,^17^ who found no difference in aerosol and spatter reduction during ultrasonic scaling with different suction types. This is likely due to a difference in sampling method (ie, Holloman et al^17^ examined bacterial counts), the distance between the operative area and sampling units, and the wider scatter that we observed. Moreover, Holloman et al^17^ measured real infection risk, whereas our study focused on measuring on aerosol generation alone. Yang et al^3^ also mentioned that simulated environments without a live patient is a limitation of many studies. However, regardless of saliva transmission and presence of patients, the efficacy of different suction should not change whether the experiment is done in the human mouth or mannequin because the procedure is the same. In fact, patients create more variables because each patient’s oral hygiene and requirements of restorative work vary. Hence, the value of standardizing the experiment and eliminating potential variables outweighs the limitation of using mannequins. Previous studies that mainly measured aerosol and splatter during ultrasonic scaling with HVE and LVE also reported significant differences between the 2 variables, with reports of a ∼90%–93% aerosol reduction.^5,25,33–35^

In SARS-CoV-2 infections, when aerosol is emitted by a person breathing and/or speaking, particles of <5 µm in diameter carry more virus than larger particles.^36^ In the present study, we considered particle volume, summed over the size range measured. Some of these particles will consist solely of irrigation or cooling water and carry no pathogens. Others will carry a proportion of the patient’s saliva and present an infection hazard. The proportion may vary with particle size and where in the oral cavity they originate. Particle size determines where in the infectee’s airway the particles deposit, which can affect infection severity. This is an area that might benefit from future research.

The effectiveness of HVE was evident for drilling as well, regardless of the number of coolant ports used and the location of teeth. The most significant finding was that HVE was effective in removing all sizes of aerosol particles measured. This is noteworthy because previous reported methodologies failed to measure small aerosol particles (0.5–10 µm). Small particles deposit in human pulmonary bronchioles and alveoli. Our study demonstrates that using either HVE or LVE significantly reduces aerosols of this particle size during drilling and scaling (Fig. 3b).^4,14,37^ Although we observed similar trends to those reported by Matys and Grzech-Lesniak,^14^ they reported a significantly lower concentration of aerosol particles, which could be explained by differences in room size, the distance at which the particle sensor was placed, and/or different settings on their air-purifier system.

One novelty of our study was the evaluation of the ‘no chip air’ aerosol-reducing function found on newer HSHs. Conventionally, aerosol exposure is managed using HVE, by decreasing handpiece speed, or by using handpieces with fewer coolant ports. Although the new ‘no chip air’ function is innovative, whether the absence of air spray could affect the pulpal temperature of the drilled tooth remains unknown. More recently, Lempel and Szalma^38^ found that effective ‘no chip air’–mediated reduction of aerosol is possible while maintaining a thermally safe environment for the tooth.

We investigated the effect of suction systems and air spray settings for dental HSHs and ultrasonic scalers on aerosol generation. Following the air sampling protocol described here, future research could involve other aerosol-related variables (eg, aerosol settling time). Although it has been reported that droplets take 30–0 minutes to settle,^12^ the time and distance variables of droplets remain unknown. The present study was conducted in an enclosed clinical room and should be repeated in an open clinical environment with single- or multiple-chair units to determine whether the safer combination of suction systems, and handpiece and scaler spray functions still apply.

In conclusion, within the current study limitations, we report several findings. The most intensive dental aerosol was generated by high-speed handpieces and ultrasonic scaling. Caution should be exercised during these procedures to minimize cross infection. For scaling, the HVE suction system was more effective in reducing aerosol generation than LVE suction or no suction at all. We recommend the new aerosol-reducing ‘no chip air’ function (if available) for cavity preparation as the function is highly effective in reducing the aerosol generated, regardless of the number of coolant ports and the location or type of tooth being prepared.
